# Fabella prevalence rate increases over 150 years, and rates of other sesamoid bones remain constant: a systematic review

**DOI:** 10.1111/joa.12994

**Published:** 2019-04-17

**Authors:** Michael A. Berthaume, Erica Di Federico, Anthony M. J. Bull

**Affiliations:** ^1^ Department of Bioengineering Imperial College London London UK

**Keywords:** fabella, Korea, prevalence rate, sesamoid bone

## Abstract

The fabella is a sesamoid bone located behind the lateral femoral condyle. It is common in non‐human mammals, but the prevalence rates in humans vary from 3 to 87%. Here, we calculate the prevalence of the fabella in a Korean population and investigate possible temporal shifts in prevalence rate. A total of 52.83% of our individuals and 44.34% of our knees had fabellae detectable by computed tomography scanning. Men and women were equally likely to have a fabella, and bilateral cases (67.86%) were more common than unilateral ones (32.14%). Fabella presence was not correlated with height or age, although our sample did not include skeletally immature individuals. Our systematic review yielded 58 studies on fabella prevalence rate from 1875–2018 which met our inclusion criteria, one of which was an outlier. Intriguingly, a Bayesian mixed effects generalized linear model revealed a temporal shift in prevalence rates, with the median prevalence rate in 2000 (31.00%) being ~ 3.5 times higher than that in 1900 (7.64%). In all four countries with studies before and after 1960, higher rates were always found after 1960. Using data from two other systematic reviews, we found no increase in prevalence rates of 10 other sesamoid bones in the human body, indicating that the increase in fabella prevalence rate is unique. Fabella presence/absence is due to a combination of genetic and environmental factors: as the prevalence rates of other sesamoid bones have not changed in the last 100 years, we postulate the increase in fabella prevalence rate is due to an environmental factor. Namely, the global increase in human height and weight (due to improved nutrition) may have increased human tibial length and muscle mass. Increases in tibial length could lead to a larger moment arm acting on the knee and on the tendons crossing it. Coupled with the increased force from a larger gastrocnemius, this could produce the mechanical stimuli necessary to initiate fabella formation and/or ossification.

## Introduction

The fabella (Latin for ‘little bean’) is a sesamoid bone located in the knee joint behind the lateral femoral condyle. Embedded in the tendon of the lateral head of the gastrocnemius muscle, it is stabilized by the fabellofibular ligament, connecting the distal insertion of the fabella to the fibular head (Minowa et al. 2004; Piyawinijwong et al. 2012; Driessen et al. 2014; Hauser et al. 2015; Kurtoğlu et al. 2015) and the posterior capsule of the knee. In rare instances, it serves as an additional origin for a muscle bundle of the popliteal muscle (Duc et al. 2004). Fabella prevalence in humans ranges from 3 to 87% (Silva et al. 2010; Zeng et al. 2012), making it a normal variant in human anatomy. The highest rates reported are in Asians and Australians, and the lowest rates in Europeans and South Americans (Minowa et al. 2004; Silva et al. 2010; Zeng et al. 2012; Hauser et al. 2015). Although its exact function is unknown, the fabella is more common in non‐human mammals (Pearson & Davin, 1921; Sarin et al. 1999), which has prompted functional and evolutionary debates about the role of the fabella in locomotion (Sarin et al. 1999; Jin et al. 2017).

Most studies reporting on prevalence rates in humans have determined the presence of the fabella through surgeries/dissections (Agathangelidis et al. 2016), X‐rays (Pancoast, 1909), computed tomography (CT) scans (Hauser et al. 2015), and magnetic resonance imaging (MRI) scans (Hedderwick et al. 2017). Ultrasound (Sekiya et al. 2002) and PET‐CT (Usmani et al. 2017) have been used to examine the fabella, but no studies have employed these methods to calculate fabella prevalence rate. Problems in calculating prevalence rates can occur depending on the method employed. For example, fabellae are sometimes so small they are difficult to detect on MRI scans, particularly if the knee is not positioned correctly (Yu et al. 1996; Ehara, 2014). Additionally, as the fabella is occasionally cartilaginous (Jin et al. 2017), its presence may not always be detected by X‐rays or CT scans. For example, a recent study on a Chinese population reported that 57.9% of the cartilaginous fabella were not visible on radiographs (Zeng et al. 2012). This highlights an issue with comparing prevalence rates between studies, as some consider only osseous fabellae, whereas others also consider cartilaginous ones. Comparing prevalence rates is further complicated as several studies do not specify whether the prevalence rates included cartilaginous with more recent ones (Hessen, 1946). This is true regardless of whether the more recent studies calculate their prevalence rates using bony or bony and cartilaginous fabellae.

Recent studies often rely on hospital archives of previously gathered X‐rays, CT scans or MRIs as a cost‐effective way of collecting data. Unfortunately, this has the potential to lead to a skewed sample, as imaging is initially done to investigate knee problems, and the presence of the fabella has been associated with several knee ailments. These include common peroneal neuropathy (Mangieri, 1973; Patel et al. 2013; Cesmebasi et al. 2016), chondromalacia (Goldenberg & Wild, 1952; Grisolia & Bartels, 1959; Robertson et al. 2004), osteoarthritis (Wolf & Bryk, 1959; Hagihara et al. 1994), popliteal artery entrapment syndrome (Ando et al. 2017), nerve palsy (Itoman et al. 1976; Takebe & Hirohata, 1981; Kubota et al. 1986; Tabira et al. 2012; Décard et al. 2017), and rheumatoid arthritis (Uchino et al. 1992). The fabella can also cause pain through dislocation (Frey et al. 1987; Franceschi et al. 2007), fracture (Sagel, 1932; Levowitz & Kletschka, 1955; Ikeuchi & Nagatsuka, 1970; Dashefsky, 1977; Woo, 1988; Marks et al. 1998; Theodorou et al. 2005; Tang et al. 2010; Heideman et al. 2011; Barreto et al. 2012; Cherrad et al. 2015; Kwee et al. 2016; Zhou et al. 2017), and generalized discomfort, a condition known as fabella syndrome (Weiner et al. 1977; Weiner & Macnab, 1982; Erichsen, 1997; Zipple et al. 2003; Segal et al. 2004; Dannawi et al. 2010; Seol et al. 2016; Kim et al. 2018; Rankin et al. 2018). As with any other joint, the interaction between the fabella and the femur can cause degenerative joint diseases, such as fabella‐femoral osteoarthritis (Urata et al. 2015).

Finally, the fabella can be problematic in cases of total knee arthroplasty (Larson & Becker, 1993; Wang, 1995; Erichsen, 1997; Segal et al. 2004; Theodorou et al. 2005; Jung et al. 2007; Hou, 2016; Kwee et al. 2016; Okano et al. 2016). The absence of an articulating groove in the back of the lateral femoral condyle, which serves to stabilize the fabella and is present in some anatomical variants (e.g. Chew et al. 2014), leads to a fabella medio‐lateral instability, causing it to painfully ‘snap’ over the replacement condyle. The reason for this pain is not known. Hou (2016) recently investigated the effects of the fabella on posterolateral pain and palsy of common peroneal nerve following total knee arthroplasty. During trials, fabellae were excised from some patients but left in others. Post‐surgery, posterolateral pain and palsy of common peroneal nerve were only observed in patients who still had fabellae. Accordingly, Hou recommended removing the fabella when knee replacement surgery is performed.

Here, we present the prevalence rate of the fabella in a population of Koreans using a randomized previously gathered dataset. As factors related to sex and length/speed of growth and development are correlated to bone formation (i.e. men are generally taller, and tall people have longer bones that are generally mechanically loaded more heavily), we investigate the effects of sex, age, and height on fabella prevalence rate. In addition, as other studies have reported higher rates for bilateral fabellae than for unilateral ones (Phukubye & Oyedele, 2011; Piyawinijwong et al. 2012; Egerci et al. 2017), we investigate whether bilateral or unilateral fabellae are more common.

To contextualize our prevalence rate results, we performed a systematic review to determine how Koreans compare with other populations, investigated possible changes in prevalence rate through time, and compared this with prevalence rates of other sesamoid bones.

## Materials and methods

### Prevalence rate

#### Sample

A **randomized sample of previously** collected CT scans, totalling 212 knees from 106 individuals (*f* = 55, *m* = 51), were investigated for the presence of the fabella (Dai et al. 2012). Scans were gathered as part of a larger project to examine human anatomy, and represent a randomized sample of Koreans. Ages of the individuals ranged from 21 to 60 years (mean/median = 52.45/55 years) and heights from 146 to 178 cm (mean/median = 160.65/160 cm; Table 1). The resolution of the scans ranged from 0.8220 × 0.8220 mm^2^ to 0.9626 × 0.9626 mm^2^ with a slice thickness of 1.0000 mm.

**Table 1:** Average and median age and heights for our sample, divided by sex. Men are taller than women

	Age (years)		Height (cm)	
	Mean ± SD	Median (Q1, Q3)	Mean ± SD	Median (Q1, Q3)
Male	50.86 ± 9.82	54 (44, 59)	165.41 ± 6.33	164 (161, 170)
Female	53.93 ± 8.07	57 (51, 60)	156.24 ± 5.08	156 (153, 160)
Total	52.45 ± 9.05	55 (47, 60)	160.65 ± 7.32	160.5 (155, 165)

CT scans prohibit the distinction between highly dense, cartilaginous and ossified fabellae, and detection of lower density, cartilaginous fabellae. Accordingly, we made no distinction between cartilaginous and bony fabellae. As it is likely that many cartilaginous fabellae are missed by CT scans, this reported prevalence rate represents a minimum rate for this sample.

#### Data collection

**Figure 1:**
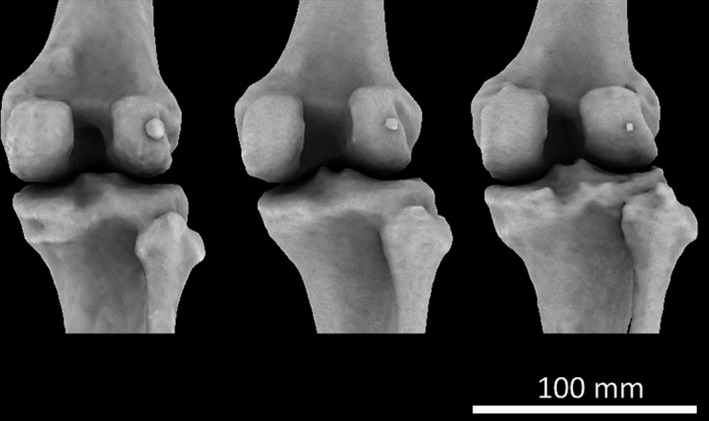
Large (left), medium (centre), and small (right) ossified fabellas in the right knees of three female subjects.

We recorded the presence/absence of the fabella on both right and left knees. Although the fabella is located behind the lateral condyle of the femur, the rest of the knee was inspected for sesamoid bones as (1) fabella presence is often correlated with the presence of other sesamoid bones (Sarin et al. 1999) and (2) some studies have reported fabellae in the medial head of the gastrocnemius (Kawashima et al. 2007; Zeng et al. 2012). Due to the resolution of the CT scans and the miniscule size of some of the fabellae (Fig. 1), fabella dimensions were not measured.

### Systematic review

#### Data sources

To complete a comprehensive literature review, the following search strategies were used for the systematic review: (1) computer search of databases and (2) review of bibliographies of all articles retrieved. Textbooks were not utilized unless they specifically came up in the computer search or bibliographies. This strategy is in accordance with Stroup et al. (2000).

#### Computer search

We searched google.scholar.co.uk for articles pertaining to the fabella in April 2018 and updated our results in October 2018. The search term *fabella* yielded 9140 results, many of which were not relevant to this study. To narrow the results, the following search terms were employed: *fabella sesamoid, fabellae sesamoid ‐fabella, fabella knee ‐sesamoid, fabellae knee –sesamoid ‐fabella, cyamella –fabella ‐fabellae, fabella incidence rate –sesamoid ‐knee, fabellae incidence rate –sesamoid –knee ‐fabella, fabella prevalence rate –sesamoid –knee ‐incidence,* and *fabellae prevalence rate ‐sesamoid ‐knee ‐fabella ‐incidence*. A hyphen before a word indicates the following word was excluded from that search, preventing the same article/citation from appearing in multiple searches.

Abstracts were reviewed first by M.A.B., and later by E.D.F. if necessary, and selected for further review if they met the following criteria: (1) the studies were on humans, (2) the studies were anatomical or medical in nature, (3) not case studies, and (4) a link was provided through which the article could be accessed. Full texts were reviewed by M.A.B.. Studies were excluded if they (1) did not report on prevalence or incidence rates based on data gathered in that study, (2) calculated rates with samples <12 knees, (3) did not report on the number of knees analysed in the study or (4) did not use a randomized sample (e.g. studies on fabella syndrome). If studies were not written in English, they were translated either by people fluent in those languages or using google translate. While imperfect, google translate worked well enough to extract the necessary data.

#### Review of bibliographies

If papers referenced other studies on prevalence rates, full texts of those studies were obtained through scholar.google.co.uk or interlibrary loan. If the original studies could not be located (as was the case with several older studies), data were extracted from the paper that referenced the original study, when possible. If not possible, the original study was excluded.

### Statistical analysis

#### Korean dataset

R and rstudio were used for statistical analyses (R Team, 2015; R Core Team, 2018). Prevalence rates for the Korean population were calculated as the percentage of knees with fabellae and individuals with fabellae. For those with fabellae, the percentage of bilateral and unilateral cases was calculated. Pearson's chi‐square tests were performed using the chisq.test function (simulate *P‐*value = TRUE, *B* = 10 000) to investigate the correlation between sex and prevalence rates. The simulate‐*P‐*value simulates datasets using Monte Carlo simulations to estimate *P*‐values for chi‐square tests. A Pearson's chi‐square test was performed with the unilateral data to investigate bilateral asymmetry. Point biserial correlations with exact *P*‐values were performed to identify the relationship between fabella presence, age, and height.

#### Systematic review

Published studies report on both knee and individual prevalence rates. We chose to transform all prevalence rates to knee prevalence rates for two reasons. First, several studies prior to 1950, reported knee and not individual prevalence rates, and it is not possible to know how many individuals were in the sample, especially considering some samples had an odd number of knees. Secondly, some studies were carried out on single legs and on whole individuals (e.g. X‐rays taken of just one knee, or only one knee was available for dissection). Studies in which this transformation could not be performed were excluded.

A Bayesian mixed effects generalized linear model was executed to investigate temporal changes in prevalence rate while accounting for the random effects of country and method for data collection using the rethinking package in R (McElreath, 2016). A logistic regression was utilized to ensure prevalence rates were between 0 and 1. The model predicts the number of fabellae present for a given sample size, allowing the regression to take study sample size into account, which varied greatly, from 12 to 2340 knees. ‘Country’ was the country in which the study was conducted, unless the study specified the race of the sample used. If more than one race was specified, each race was treated as an individual data point (Miaśkiewicz & Partyka, 1984). ‘Method’ was the method for data collection, either X‐ray, CT scans, MRI or anatomical dissection. If more than one method was used, each method was treated as an individual data point (Chew et al. 2014; Hedderwick et al. 2017).

The Bayesian model followed protocol set out by McElreath (2016). The map2stan function was used to create a binomial distribution using the number of knees and fabellae in the published studies. The probability that a fabella would be present was defined as follows:

$$\text{logit}\left(\text{probability}\right)=\alpha +\alpha_{\mathrm{country}}+\alpha_{\mathrm{method}}+\beta *\text{Year}$$

where broad, weakly regularizing priors were used for the fixed (α, β) and random (α_country_, α_method_) effects (see Data S1 for further details). Markov‐chain Monte Carlo (MCMC) estimation was used to estimate the posterior probability distribution (4 chains, 10 000 iterations, 1000 iterations warmup).

To determine whether there were any outliers, a Pearson's linear regression was run between the natural log of knee and fabella count. A log transformation was used, as the density plot of study sample sizes were non‐normally distributed. If any study had an unusually high or low variance (i.e. an unusually high or low number of fabellae for that sample size), it was considered an outlier and removed from further analyses. After outliers were removed, the missing data concerning ‘method for data collection’ was imputed using the mice package in R (van Buuren & Groothuis‐Oudshoorn, 2011) . Two methods were used to impute the data, ‘pmm’ and ‘lmer,’ to create 20 imputed datasets (10/method). The consensus results were used for further analysis.

## Results and Discussion

### Korean dataset

Fabellae were present in 56/106 individuals (52.83%) and 94/212 knees (44.34%). All fabellae were located in the lateral heads of the gastrocnemius: other than the patella, no other sesamoid bones were observed in the knees. Of the 56 individuals with fabellae, bilateral cases were more prevalent than unilateral ones (bilateral = 38/56, unilateral = 18/56, χ^2^ = 7.1429, *P* = 0.0107). Of the 32 female cases, bilateral cases were as prevalent as unilateral (bilateral = 20/32, unilateral = 12/32, χ^2^ = 2, *P* = 0.2110), but of the 24 male cases, bilateral cases were more prevalent than unilateral ones (bilateral = 18/24, unilateral = 6/24, χ^2^ = 6, *P* = 0.0238). Our prevalence rate of 67.86% falls slightly below the prevalence rate of ~ 80% bilateral cases reported by other studies (Sutro et al. 1935; Pritchett, 1984), but rates of ~ 50–66% have been reported (Houghton‐Allen, 2001; Phukubye & Oyedele, 2011; Piyawinijwong et al. 2012; Egerci et al. 2017). The relatively high prevalence rates of fabellae in this sample were comparable to those reported in other Asian samples (e.g. 28.50–86.69% in Chinese and 15.29–85.85% in Japanese samples; Table 2).

**Table 2:** Results from the systematic review. Source column indicates the source the information was retrieved from

Author	Year	Source	Method	Country	No. of knees	No. of fabellae	Reported prevalence rate (*100)	Adjusted rate (*100)
Gruber	1875	1	Anatomical	Russian	2340	400	17.09	17.09
Ost	1877	1	Anatomical	Switzerland	30	5	16.67	16.67
Pfitzner	1892	1	Anatomical	Germany^a^	291	30	10.31	10.31
Parsons and Keith	1897	2	Unknown	UK	287	81	28.22	28.22
Pancoast^b^	1909	3	X‐ray	USA	–	–	–	–
Fischer	1912	1	X‐ray	Germany	410	72^c^	17.6	17.6
Frey	1913	1	Anatomical	Switzerland	113	15	13.3	13.3
Sugiyama	1914	1	Unknown	Japan	75	36	48	48
Pichler	1918	4	Unknown	Austria	100	8	8	8
Hanamuro	1927	1	X‐ray	China	400	114	28.5	28.5
Pick	1927	1	X‐ray	Germany	300	22	7.33	7.33
Rothe	1927	1	X‐ray	Germany	600	86	14.33	14.33
Sonntag	1927	1	X‐ray	Germany	1000	145	14.5^d^	14.5
Yano	1928	5	Anatomical	Japan	165	44	26.67	26.67
Heydemann	1929	1	X‐ray	Germany	427	58	13.58	13.58
Greifenstein	1930	1	X‐ray	Germany	100	16	16	16
Haussecker	1930	1	X‐ray	Germany	280	32	11.43	11.43
Ooi (Oi?)^e^	1930	6	Unknown	Japan	80	25	31.25	31.25
Sommer	1930	1	X‐ray	Germany	200	25	12.5	12.5
Sonntag	1930	1	X‐ray	Germany	690	119	17.25	17.25
Siina	1931	1	Unknown	Japan^f^	10	4	40	40
Mikami	1932	1	Unknown	Japan	510	78	15.29	15.29
Bircher and Oberholzer	1934	7	X‐ray	Switzerland	700	46	6.6	6.6
Chung	1934	1	Anatomical	Korea	348	104	29.89	29.89
Kobayashi	1934	1	X‐ray	Japan^g^	292	83^h^	28.42	22.9
Kitahara	1935	8	X‐ray	Taiwan	100	17	17	13.6
Sutro et al.	1935	1	X‐ray	USA	806	97^i^	12.03	12.03
Hessen	1946	9	X‐ray	Sweden	942	154	16.35	16.35
Lungmuss	1954	1	X‐ray	Germany	1000	192	19.2	19.2
Schonbauer	1956	10	X‐ray	Austria	1000	122	12.2	12.2
Kojima	1958	11	Anatomical	Japan	152	53	34.87	34.87
Falk	1963	12	X‐ray	USA	1023	132	12.3	12.3
Kaneko	1966	6	Anatomical	Japan	150	63	42	42
Johnson & Brogdon	1982	12	X‐ray	USA	1304	128	9.82	9.82
Hukuda et al.,^j^	1983	13	X‐ray	Japan	–	–	–	–
Miaskieqicz & Partyka	1984	13	X‐ray	Poland	52	8	15.38	15.38
Miaskieqicz & Partyka	1984	13	X‐ray	Vietnam	34	8	23.53	23.53
Miaskieqicz & Partyka	1984	14	X‐ray	West Africa	102	10	9.8	9.8
Sudasna & Harnsiriwattanagit	1990	15	Anatomical	Thailand	50	34	68	68
Chihlas et al.	1993	16	Anatomical	USA	66	18^k^	27.27	27.27
Hagihara, et al.,	1993	17	Unknown	Japan	302	164	54.3	54.3
Terry & LaPrade	1996	18	X‐ray	USA	25	5	20	20
Yu et al.,	1966	19	MRI	USA	100	19	19	19
De Maeseneer et al.	2001	20	MRI	Belgium	122	32	26.23	26.23
Munshi et al.	2003	21	Anatomical	USA	1	1	100	100
Munshi et al.	2003	21	MRI	USA	7	4	57.14	57.14
Minowa et al.	2004	22	Anatomical	Japan	212	182	85.85	85.85
Kawashima et al.	2007	23	Anatomical	Japan	75	43^l^	57.33	57.33
Rahemm et al.	2007	24	Anatomical	Ireland	22	2	9.09	9.09
Lencina	2007	25	X‐ray	Argentina	217	45	20.73	20.73
Lencina	2007	25	Anatomical	Argentina	22	3	13.64	13.64
Silva et al.	2010	26	Anatomical	Brazil	62	2	3.23	3.23
Phukubye, Oyedele	2011	27	Anatomical	South Africa	102	18	17.65	17.65
Zeng et al.	2012	28	X‐ray	South Africa	146	22	15.07	15.07
Kato et al.	2012	29	X‐ray	Macedonia	60	8	13.33	13.33
Tabira et al.	2012	30	Anatomical	Japan	150	122	81.33	81.33
Dodevski et al.	2012	31	Anatomical	Thailand	372	144	38.71	38.71
Damon	2012	32	Anatomical	Japan	102	70	68.63	68.63
Piyawinijwong et al.	2012	33	Anatomical	China	61	53^m^	86.89	86.89
Chew et al.^n^	2014	34	X‐ray	Asians	–	–	–	–
Chew et al.^n^	2014	34	MRI	Asians	–	–	–	–
Hauser et al.	2015	35	Anatomical	Central Europe	400	105	26.25	26.25
Upasna et al.	2016	36	Anatomical	India	40	5	12.5	12.5
Mohite et al.	2016	37	Anatomical	Indian	60	8	13.33	13.33
Jin et al.	2017	38	X‐ray	Turkey	1000	190	19	19
Ghimire et al.	2017	39	X‐ray	Nepal	155	19	12.26	12.26
Hedderwick et al.	2017	40	MRI	New Zealand	25	14	56	56
Hedderwick et al.	2017	40	Anatomical	New Zealand	28	8	28.57	28.57
Egerci et al.	2017	41	Anatomical	Japan	16	9	56.25	56.25
Corvalan et al.	2018	42	Anatomical	Australia	111	63	56.76	56.76
Ortega & Olave	2018	43	X‐ray	Chile	400	125	31.25	31.25
Tatagari et al.	2018	44	Anatomical	USA	182	52	28.57	28.57
This study	2018		CT scans	Korea	212	94	44.34	44.34

a Location: Alsace: Germany at the time, now France.

b 67/529 individuals had fabellae.

c Estimated 72 fabellae based on an prevalence rate of 17.6%.

d Estimated 145 fabellae based on an prevalence rate of 14.5%.

e When translated from characters, the spelling could be Ooi or Oi.

f Reported location was Aino, taken from Hessen (1946).

g Reported location was Hokuriku‐Japaner.

h Estimated 83 fabellae based on an prevalence rate of 28.42%.

i Hessen had 96. Sutro had 81 patients with at least one fabella. 106 patients had roentgenograms of both knees, 16 were bilateral. Therefore, there are 97 fabellae in total.

j 11/31 individuals had fabellae.

k Estimated 18 fabellae based on an prevalence rate of 27%.

l Reports on fabellae in medial head – ignored here, as it is unusually high, particularly given the lack of medial fabellae in other studies.

m Reports a couple of medial fabellae – not possible to tease them out, prevalence rate may be too high.

n Prevalence rate of 31.25% (25/80) for individual. Unknown if one or two knees were inspected per individual.

There were no differences between males and females in terms of knee (*f* = 52/110, *m* = 42/102, χ^2^ = 0.7970, *P* = 0.4059) or individual (*f* = 32/55, *m* = 24/51, χ^2^ = 1.3138, *P* = 0.3341) prevalence rates (Table 3). Both men and women were equally likely to have bilateral (*f* = 20/55, *m* = 18/51, χ^2^ = 0.0132, *P* = 1) or unilateral (*f* = 12/55, *m* = 6/51, χ^2^ = 1.8972, *P* = 0.2087) fabellae. These results are in agreement with other fabella studies, in which no sex‐based differences in fabella presence/absence were observed (Parsons & Keith, 1897; Chew et al. 2014; Ortega & Olave, 2018). Within unilateral cases, fabellae were equally likely to be present in the right or left knee (right = 8/18, left = 10/18, χ^2^ = 0.222, *P* = 0.8177).

**Table 3:** Prevalence rates broken down by subcategories (individuals, knees) and sex

	Knees	Individuals	Percentage bilateral	Percentage unilateral
Male	41.18% (42/102)	47.06% (24/51)	75.00% (18/24)	25.00% (6/24)
Female	47.27% (52/110)	58.18% (32/55)	62.50% (20/32)	37.50% (12/32)
Total	44.34% (94/212)	52.83% (56/106)	67.86% (38/56)	32.14% (18/56)

There were no sex‐based differences. Of the 56 individual cases, bilateral cases were significantly more prevalent than unilateral ones. Bilateral cases were more prevalent than unilateral in males (*n* = 24), but there was no difference in females (*n* = 32). Within unilateral cases, fabellae were equally likely to be present in the right or left knee. There were no differences between the sexes (see text for test statistics and *P*‐values).

Height was not correlated to individual prevalence rate (rpbi = −0.0245, *t* = −0.2502, df = 104, *P* = 0.8029), or the likelihood of having bilateral (rpbi = 0.0574, *t* = 0.5867, df = 104, *P* = 0.5587) or unilateral (rpbi = −0.106, *t* = −1.0869, df = 104, *P* = 0.2796) fabellae (Table 4). These results are supported by the substantiated knowledge that the number of ossification centres is not correlated to adult height in humans.

**Table 4:** Results showing no correlation between height/age and prevalence of fabellae in individuals, or the percentage of bilateral/unilateral cases (i.e. are taller individuals more or less likely to have bilateral fabellae?). Degrees of freedom were all 104, *P*‐values were all > 0.25. (r = correlation coefficient; *t* = test statistic)

	Individuals		Percentage bilateral		Percentage unilateral	
	*r*	*t*	*r*	*t*	*r*	*t*
Height	−0.0245	−0.2502	0.0574	5867	−0.106	−1.0869
Age	0.0601	0.6143	−0.0136	−0.1384	0.0973	0.9967

Similarly, individual prevalence rate was not correlated to age (rpbi = 0.0601, *t* = 0.6143, df = 104, *P* = 0.5404), or the likelihood of having bilateral (rpbi = −0.0136, *t* = −0.1384, df = 104, *P* = 0.8902) or unilateral (rpbi = 0.0973, *t* = 0.9967, df = 104, *P* = 0.3212) fabellae. This is not surprising as all individuals in this study were skeletally mature (age 21+ years), and new ossifications do not typically occur during adulthood. Three studies on human foetuses reported the fabella to be common (Jin et al. 2017), rare (Minowa et al. 2005) or completely absent (Oransky et al. 1989) at early stages of development, suggesting fabella initiation time is variable in humans.

One study investigating prevalence rates in a Japanese population identified a correlation between fabella prevalence rate and age, finding a lower prevalence rate in younger (< 50 years, 31%) than older individuals (> 50 years, 47%) (Kato et al. 2012). In our dataset, individuals < 50 years old were no more or less likely to have a fabella than were individuals > 50 years old (younger = 23/94, older = 35/118, χ^2^ = 0.7099, *P* = 4448).

**Figure 2:**
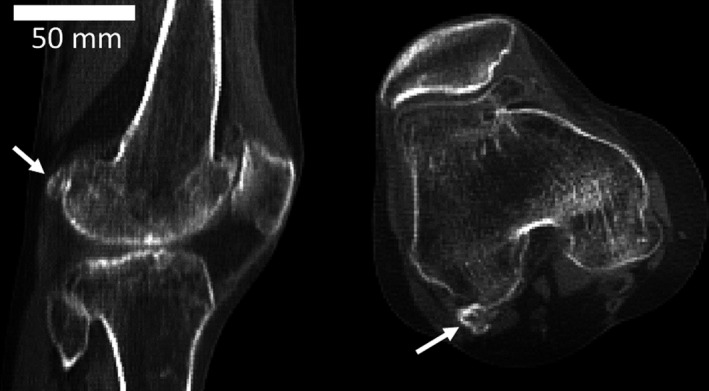
Lateral (left) and superior (right) views of the fabella (white arrow).

In this study, fabellae ranged in size from small (just a few pixels) to large (Fig. 1). In general, fabellae did not appear to articulate with the lateral femoral condyle. However, the CT scans were acquired postmortem, and soft tissues were severely deformed in most individuals, making it possible that some fabellae would have articulated with the condyle in life but were separated in death. Some large fabellae were still articulated with the posterior surface of the lateral femoral condyle, the most drastic of which was observed in female 005 (Fig. 2), which shows a large articulating surface in the femur.

### Systematic review

Our searches revealed 2631 abstracts on fabella prevalence rates between 1875 and 2018, written in seven languages (English, German, French, Spanish, Italian, Japanese, and Chinese). It should be noted that the authors are not confident they identified all non‐English studies, as it is possible non‐English studies exist without translated titles/abstracts and as such were not detected by our search terms. Also, we are not confident we identified all studies < 75 years old, as we discovered some in bibliographies that did not come up in our scholar.google.co.uk searches.

A total of 185 full‐text articles/conference proceedings were reviewed, 66 of which reported on fabella prevalence rates. Of the 66 studies, five were discarded from further analysis as they did not fit the inclusion criteria. Pancoast (1909) and Hukuda et al. (1983) reported that 67/529 and 11/31 individuals from the USA and Japan, respectively, had fabellae, but these could not be transformed into a knee prevalence rate (Pancoast, 1909; Hukuda et al. 1983). Chew et al. (2014) reported on a prevalence rate of 31.25% (25/80) in ‘Asians’, but we could not determine whether this was an individual or knee rate (Chew et al. 2014). Siina (1931), taken from Table 1 (tabelle I) in Hessen (1946), and Munshi et al. (2003), had a sample sizes of 10 and 8 knees, respectively (Munshi et al. 2003). Finally, three studies claimed to have data on fabella presence/absence, but the data were not present, at least not in the versions of the papers we had access to (Nishimura & Shimizu, 1963; Orzincolo et al. 1987; Osti et al. 2013). Our final analysis included 21 676 knees and represented studies done in 27 countries. It should be noted that Taiwan was part of Japan from 1895 to 1945, at the time of studies of Kitahara (1935) and Hanamuro (1927). According to Hessen (1946), Kitahara's (1935) sample was ‘Formosawilde’, indicating it consisted of the natives of Taiwan. As such, we have classified this sample as being from Taiwan, even though no such political entity existed at the time. According to Hessen (1946), Hanamuro (1927) included individuals from Formosa as well, but classified them as ‘Formosa‐Chinesen’, indicating they were immigrants from mainland China into Taiwan. As such, we classified their sample as being from China. A summary of prevalence rates reported in the literature can be found in Table 2.

**Figure 3:**
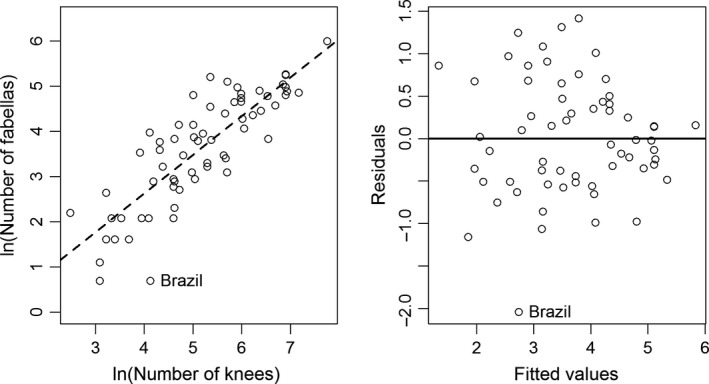
Plot of the natural log of sample size (number of knees) and number of fabellas for the 57 studies considered for this analysis. A Pearson's correlation revealed a statistically significant relationship between the two variables (*y *= 0.82350 * x −0.60879; *t*‐value = 11.149, *P *= 2.96e‐16), with an intercept that is not statistically different from zero (*t*‐value = −1.541, *P* = 0.129). The data for Brazil (Silva et al., 2010) represent an outlier for this dataset.

We identified one outlier in our dataset (Fig. 3), as the number of fabellae (*n =* 2) was exceptionally low for that number of knees (*n* = 62). This is not to say the data are incorrect, only that it is an outlier from the other 56 studies, and thus was excluded from further analyses.

There were five studies for which the method remained ‘unknown’, either because the method was not mentioned in the study or we were not able to obtain the original study and identify the method. We assumed Parsons & Keith (1897) used anatomical dissections, as the X‐ray was invented in 1895, making it unlikely they used X‐rays to collect their data. For the four other studies, all imputed datasets yielded consistent results for Sugiyama (1914), Ooi/Oi (1930), and Mikami (1932), classifying the first two as anatomical dissections and the third as X‐ray. According to the imputed data, Pichler (1918) was categorized as X‐ray 15/20 times, MRI 3/20 times, and CT 2/20 times. As MRI and CT scanners were not invented in 1918, we assume Pichler used X‐rays to collect their data.

**Figure 4:**
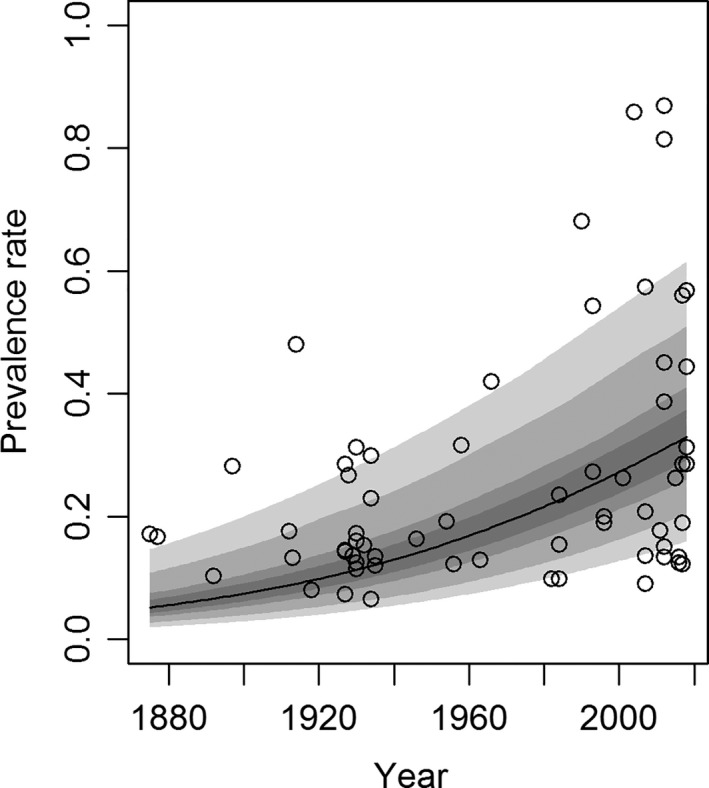
There is a statistically significant relationship between prevalence rate and time, with people being, on average, nearly 3.5 times more likely to have a fabella in 2018 than in 1918. The confidence intervals are, from widest to narrowest, 99, 95, 75, and 50%. The raw data used to create this figure are available in the Table S2.

The logistic regression revealed a strong increase in prevalence rates through time (*P*_slope_ < 0.01, *P*_intercept_ < 0.01; Fig. 4). The r code and raw data used to conduct the analysis are available in the Data S1 and Table S1. Assuming median random and fixed effects, the results show that:

$$\text{logit}\left(\text{Prevalence}\right)=-33.3390+\left(1.6314*10^{-2}\right)*\text{Year}$$

Interestingly, recent studies show a higher variance in prevalence rates compared with older studies. This is because there is an increase in maximum prevalence rates, with no real increase in minimum prevalence rates, causing a larger spread of the data. Although different populations were examined before and after 1960, and a genetic component may be involved in population‐related fabella prevalence rates (Sarin et al. 1999), the authors are confident that the observed increase in fabella prevalence rates is not affected by these factors, as described below.

**Figure 5:**
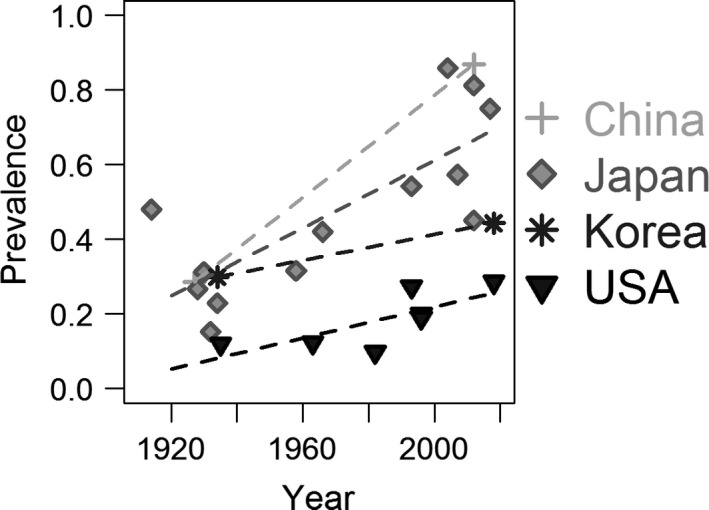
Four countries (China, Japan, Korea, and USA) had prevalence rates reported both before and after 1960. For China and Korea, there was only one study before and one study after 1960, and the lines connect these studies. For the USA and Japan, there were several, and Pearson's linear regressions were run. There is no statistically significant relationship in the USA (*P *= 0.0793), but there is a significant relationship in Japan (prevalence rates = 0.5064 * year −947.9; *P *= 2.25e‐4).

Prevalence rates were reported in four countries both before and after 1960: China, Japan, Korea, and USA. For China and Korea, there was one study before and one study after 1960; in both countries, the more recent study had a higher prevalence rate (Fig. 5). For USA and Japan, there were several studies both before and after 1960, and Pearson's linear regressions revealed positive relationships between prevalence rate and time in both countries. As there were relatively few studies in each country, we chose simpler Pearson's linear regressions in lieu of binomial mixed effect models to provide a visualization of the average change in prevalence rate over time. As random effects were ignored, little faith should be put in the regression equations and their *P*‐values (Fig. 5). Although it is not possible to hold genetics constant between the older and newer studies, particularly in countries that have large levels of genetic diversity, such as USA, this evidence supports the idea that the increase in prevalence rates is not a by‐product of different populations being used in studies before and after 1960.

Why would there be an increase in fabella prevalence rate over time? Skeletal phenotypes result from a combination of genetic and environmental factors. Although fabella formation appears to have a genetic component, it is improbable a genetic mutation is responsible for the worldwide increase in prevalence rates; the probability of a mutation occurring in *Homo sapiens* and spreading throughout the entire species in the past 100 years is an unprecedented and unlikely scenario.

Environmentally, it is possible that the increase in prevalence rates could be due to a hormonal or epigenetic shift. Since the mid‐20th century, there has been a marked increase in plastic usage (Zalasiewicz et al. 2016), and plastics are known to have deleterious effects on growth and development. For example, several chemicals found in plastics are known to disrupt hormonal pathways in vertebrates and other animals. It is therefore possible that plastics could have affected human skeletal growth and development, and be responsible for the increase in fabella prevalence rates. If a hormonal or epigenetic pathway were responsible, it is reasonable to assume the effects would be systematic, influencing all the sesamoid bones in the human body.

To test this idea, we investigated temporal changes in prevalence rates in other sesamoid bones in the human body. We identified two systematic reviews investigating sesamoid bone prevalence rates in the human hand (Yammine, 2014) and foot (Yammine, 2015) with data from 1892 onwards. Using these reviews, we investigated temporal changes in prevalence rate in six sesamoid bones in the hand and four sesamoid bones in the foot.

**Figure 6:**
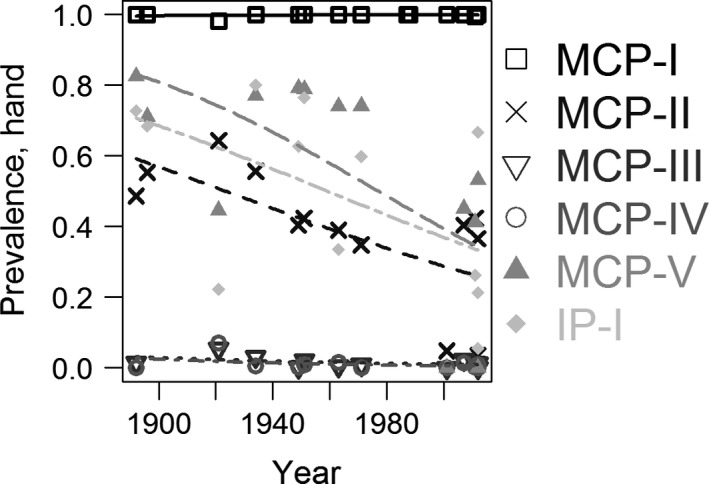
Temporal changes in six sesamoid bone in the hand: the sesamoid bones at the metacarpophalangeal (MCP) joint of the first (MCP‐I), second (MCP‐II), third (MCP‐III), fourth (MCP‐IV), and fifth (MCP‐V) fingers, and at the interphalangeal joint of the first finger (IP‐I). Data from table 2 in Yammine (2014) (*n* = 16 studies). Unlike with the fabella, there was no correlation between hand sesamoid bone prevalence and time (Table 5).

**Figure 7:**
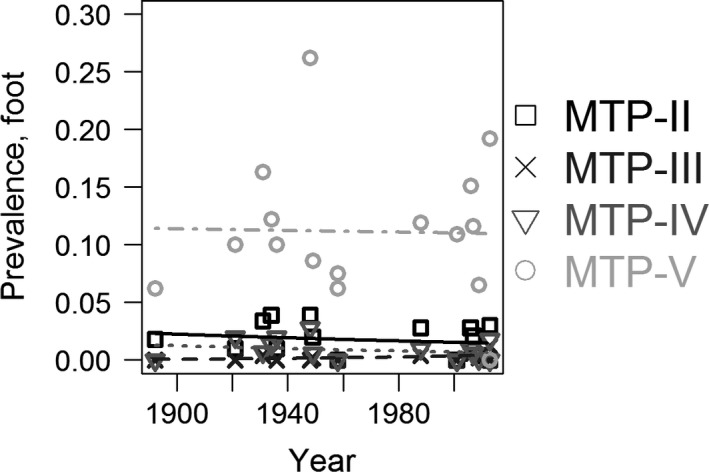
Temporal changes in four sesamoid bone in the foot: the sesamoid bones at the metatarsophalangeal (MTP) joint second (MTP‐II), third (MTP‐III), fourth (MTP‐IV), and fifth (MTP‐V) toes. Data from table 6 in Yammine (2015) (*n* = 16 studies). Similar to the sesamoid bones in the hand, there was no correlation between foot sesamoid bone prevalence and time (Table 6).

Due to the low number of studies investigating prevalence rates for these bones (16 across 120 years for the hand and 16 across 121 years for the foot), we ran binomial regressions without random effects using the glm function in R to investigate possible temporal changes. Our analyses revealed there were no temporal changes in sesamoid bone prevalence rates in either the hand or the foot (Tables 5 and 6; Figs 6 and 7). These results imply the increase in fabella prevalence rate does not have a hormonal or epigenetic origin, and the increase in fabella prevalence rate is unique.

**Table 5:** Results from binomial regressions testing the relationship between time and prevalence rates of six sesamoid bones in the hand

	*P*‐value	*Z*‐value	Degrees of freedom
MCP‐I	0.925	0.094	13
MCP‐II	0.400	−0.842	11
MCP‐III	0.855	−0.183	10
MCP‐IV	0.837	−0.205	10
MCP‐V	0.219	−1.229	11
IP‐I	0.363	−0.91	9

Data taken from Table 2 in Yammine (2014). Although Yammine (2014) reported differences in prevalence due to sex and race, all data were pooled here, as there were only 16 studies stretching over 120 years. Prevalence rates were given per hand. In cases where ulnar and radial sesamoid bones were reported separately, the higher value was used, as it was not possible to determine whether the sesamoid bones were always from the same or different individuals. *Z*‐value = test statistic. A Bonferroni‐corrected *P*‐value of 0.00833 (*P *= 0.05/6) shows a lack of any statistically significant trends.

**Table 6:** Results from binomial regressions testing the relationship between time and prevalence rates of four sesamoid bones in the feet

	*P*‐value	*Z*‐value	Degrees of freedom
MTP‐II	0.939	−0.077	14
MTP‐III	0.101	0.920	14
MTP‐IV	0.937	−0.079	14
MTP‐V	0.986	−0.017	14

Data taken from Table 6 in Yammine (2015): data on the hallux (Table 2) were not analysed because they were highly mixed. Similar to the data with the sesamoid bones in the data, all data were pooled here, as there were only 16 studies stretching over 121 years. Prevalence rates were given per foot. In cases where tibial and ulnar sesamoid bones were reported separately, the higher value was used, as it was not possible to determine if the sesamoid bones were always from the same or different individuals. *Z*‐value = test statistic. A Bonferroni‐corrected *P*‐value of 0.0125 (*P *= 0.05/4) shows a lack of any statistically significant trends.

Sesamoid bones form in areas of high mechanical stimuli, such as pressure, friction or stress (Sarin & Carter, 2000), and act to modify/reduce pressure, friction or stress. It is therefore possible that some change in mechanical loading could have caused an increase in fabella prevalence rate. Differences in loading could be due to differences in kinematics or muscle mass/bone lengths. We do not believe the differences are due to kinematics for the following reasons. First, it is unlikely that all humans, worldwide, have begun to move their lower limbs in a consistently different manner in the last 100 years. Secondly, there appears to be no correlation between magnitude of mechanical loading over one's lifetime and fabella presence in people today, with fabellae being found in both active individuals, such as non‐professional (Dashefsky, 1977; Kuur, 1986) and Olympic level athletes (Zenteno et al. 2010), and inactive individuals, such as foetuses (Minowa et al. 2005; Jin et al. 2017) and the elderly (Laird, 1991; Ando et al. 2017). Finally, unlike in other mammals, the fabella likely offers no significant mechanical advantage in humans, as when excised (common practice to address fabella syndrome), no ill mechanical effects are observed (Weiner & Macnab, 1982; Zenteno et al. 2010; Agathangelidis et al. 2016; Okano et al. 2016). This implies there may be no significant mechanical, evolutionary advantage to having a fabella (Sarin et al. 1999).

It is, however, possible global changes in muscle mass/bone lengths could be responsible. Worldwide, there has been a general increase in dietary quality and nutrition over the last 100 years, which has allowed humans to come much closer to achieving their genetic potential.^1^ This means people are taller, weigh more, and have bigger muscles today than they did 100 years ago. Increases in tibial length could lead to a larger moment arm acting on the knee and on the tendons crossing it. Coupled with the increased force from a larger gastrocnemius, this could produce the mechanical stimuli necessary to initiate fabella formation and/or ossification. However, these factors do not explain the high prevalence of cartilaginous fabellae in foetuses, or why there was no relationship between presence and height in our sample.

Lastly, it is possible there is no shift in fabella prevalence rate, but the increase in prevalence rates is due to a change in fabella identification, where fabellae that were being previously ignored are now being identified. We believe this is highly unlikely for two reasons. First, there were no other changes in the prevalence of sesamoid bones in the hand or foot, and if there was a change in sesamoid bone identification protocol, it would likely not be isolated to the fabella. Secondly, the inclusion of X‐ray and CT scans to determine prevalence rates in recent studies should lead to a decrease, not an increase, in prevalence rates through time, as cartilaginous fabellae, which may or may not have been included in previous studies, cannot be detected by X‐rays and CT scans.

In this study, we investigated the prevalence rate of the fabella in a Korean population using published CT scans. Our prevalence rate of 52.83 and 44.34% for individuals and knees, respectively, falls within the range of those reported in the literature and shows an increase in fabella prevalence in Koreans over the past 80 years. In addition, we found bilateral fabellae to be more common than unilateral ones, there were no sex differences in prevalence rates, and presence of a fabella was uncorrelated with height and age. We also found a significant increase in fabella prevalence rates through time, but we are unsure why this has occurred and why there has not been an increase in other sesamoid bones in the human body during the same time span.

## Author contributions

M.A.B., E.D.F., and A.M.J.B. conceived and designed the project. M.A.B. and E.D.F. acquired/analysed the data and performed the systematic review. M.A.B., E.D.F., and A.M.J.B. wrote/edited the manuscript and approved the final version of this article.

## Supporting information


**Data S1.** R code use for systematic review.

**Table S1.** Raw data used for Bayesian analysis.

**Table S2.** Median prevalence rates with confidence intervals.

## Note

1 The term ‘genetic potential’ often refers to the idea that humans have a genetically determined upper limit to their adult stature and anthropometric dimensions (Bogin, 2006). Although this comes dangerously close to supporting the concept of genetic determinism, we are not using it in that manner here.

## References

[joa12994-bib-0001] Agathangelidis F , Vampertzis T , Gkouliopoulou E , et al. (2016) Symptomatic enlarged fabella. BMJ Case Rep 2016, bcr2016218085. 10.1136/bcr-2016-218085 PMC5128986 27807024

[joa12994-bib-0002] Ando Y , Miyamoto Y , Tokimura F , et al. (2017) A case report on a very rare variant of popliteal artery entrapment syndrome due to an enlarged fabella associated with severe knee osteoarthritis. J Orthop Sci 22, 164–168. 26740435 10.1016/j.jos.2015.06.025

[joa12994-bib-0003] Barreto ARF , Chagas‐Neto FA , Crema MD , et al. (2012) Fracture of the fabella: a rare injury in knee trauma. J Radiol Case Rep 2012, 390150. 10.1155/2012/390150 PMC3508535 23213592

[joa12994-bib-0004] Bircher E , Oberholzer J (1934) Die Kniegelenkkapsel im Pneumoradiographie‐Bilde. Acta Radiol 15, 452–466.

[joa12994-bib-0005] Bogin B (2006) Patterns of Human Growth. 2nd edn Cambridge, UK: Cambridge University Press.

[joa12994-bib-0006] van Buuren S , Groothuis‐Oudshoorn K (2011) mice : multivariate imputation by chained equations in R. J Stat Softw 45, 1–67.

[joa12994-bib-0007] Cesmebasi A , Spinner RJ , Smith J , et al. (2016) Role of sonography in the diagnosis and treatment of common peroneal neuropathy secondary to fabellae. J Ultrasound Med 35, 441–447. 26782165 10.7863/ultra.15.04003

[joa12994-bib-0008] Cherrad T , Louaste J , Bousbaä H , et al. (2015) Fracture of the fabella: an uncommon injury in knee. Case Rep Orthop 2015, 1–3. 10.1155/2015/396710 PMC4584060 26448891

[joa12994-bib-0009] Chew CP , Lee KH , Koh JSB , et al. (2014) Incidence and radiological characteristics of fabellae in an Asian population. Singapore Med J 55, 198–201. 24763835 10.11622/smedj.2014052 PMC4291947

[joa12994-bib-0010] Chihlas CN , Ladocsi LT , Sholley MM , et al. (1993) Position of the fabella relative to the path of the common peroneal nerve across the lateral head of the gastrocnemius muscle. Clin Anat 6, 163–166.

[joa12994-bib-0011] Corvalan C , Tang C , Robinson M (2018) Fabella and cyamella of the human knee joint discovery by dissection and ultrasound examination. Eur J Anat 22, 103–109.

[joa12994-bib-0012] Dai J‐X , Chung MS , Qu R‐M , et al. (2012) The Visible Human Projects in Korea and China with improved images and diverse applications. Surg Radiol Anat 34, 527–534. 22402591 10.1007/s00276-012-0945-8

[joa12994-bib-0013] Damon CA (2012) The role of plain film radiography in the diagnosis and management of knee pain. Masters thesis. Department of Chiropractic Durban University of Technology, 68.

[joa12994-bib-0014] Dannawi Z , Khanduja V , Vemulapalli K , et al. (2010) Arthroscopic excision of the fabella – a report of two cases. J Knee Surg 20, 299–301. 10.1055/s-0030-1248063 17993073

[joa12994-bib-0015] Dashefsky J (1977) Fracture of the fabella: a case report. J Bone Joint Surg 59, 698. 873972

[joa12994-bib-0016] De Maeseneer M , Shahabpour M , Vanderdood K , et al. (2001) Posterolateral supporting structures of the knee: findings on anatomic dissection, anatomic slices and MR images. Eur Radiol 11, 2170–2177. 11702156 10.1007/s003300100983

[joa12994-bib-0017] Décard BF , Nagy S , Garcia M , et al. (2017) An unusual case of bilateral peroneal palsies due to fabellae. Neurology 88, 918. 28242848 10.1212/WNL.0000000000003666

[joa12994-bib-0018] Dodevski A , Lazarova‐Tosovska D , Zhivadinovik J , et al. (2012) Clinical significance of the fabella. Rentgenol Radiol 51, 258.

[joa12994-bib-0019] Driessen A , Balke M , Offerhaus C , et al. (2014) The fabella syndrome – a rare cause of posterolateral knee pain: a review of the literature and two case reports. BMC Musculoskelet Disord 15, 100. 24666711 10.1186/1471-2474-15-100 PMC3987160

[joa12994-bib-0020] Duc SR , Wentz KU , Kach KP , et al. (2004) First report of an accessory popliteal muscle: detection with MRI. Skeletal Radiol 33, 429–431. 15127245 10.1007/s00256-004-0775-9

[joa12994-bib-0021] Egerci OF , Kose O , Turan A , et al. (2017) Prevalence and distribution of the fabella: a radiographic study in Turkish subjects. Folia Morphol 76, 478–483. 10.5603/FM.a2016.0080 28026849

[joa12994-bib-0022] Ehara S (2014) Potentially symptomatic fabella: MR imaging review. Jpn J Radiol 32, 1–5. 24158650 10.1007/s11604-013-0253-1

[joa12994-bib-0023] Erichsen H (1997) Bilateral fabellar impingement after knee replacement‐a case report. Acta Orthop Scand 68, 403. 9310050 10.3109/17453679708996187

[joa12994-bib-0024] Falk GD (1963) Radiographic observations of the incidence of fabella. Bull Hosp Jt Dis 24, 127–129. 14173207

[joa12994-bib-0025] Franceschi F , Longo UG , Ruzzini L , et al. (2007) Dislocation of an enlarged fabella as uncommon cause of knee pain: a case report. Knee 14, 330–332. 17490883 10.1016/j.knee.2007.03.007

[joa12994-bib-0026] Frey C , Bjorkengen A , Sartoris D , et al. (1987) Knee dysfunction secondary to dislocation of the fabella. Clin Orthop Relat Res 222, 223–227. 3621725

[joa12994-bib-0027] Ghimire I , Maharjan S , Pokharel GB , et al. (2017) Evaluation of occurrence of sesamoid bones in the lower extremity radiographs.

[joa12994-bib-0028] Goldenberg RR , Wild EL (1952) Chondromalacia fabellae. J Bone Joint Surg 34, 688–690. 14946223

[joa12994-bib-0029] Grisolia A , Bartels WW (1959) Chondromalacia of the fabella. Am J Surg 98, 760–761. 13829439 10.1016/0002-9610(59)90510-0

[joa12994-bib-0030] Hagihara H , Nakaie K , Kishikawa Y , et al. (1993) Incidence and size of fabella in osteoarthrosis of the knee. Orthop Traumatol 42, 995–997.

[joa12994-bib-0031] Hagihara H , Nakaie K , Kishikawa Y , et al. (1994) Knee dysfunction caused fabella. Orthop & Traumatol 43, 542–544.

[joa12994-bib-0032] Hauser NH , Hoechel S , Toranelli M , et al. (2015) Functional and structural details about the fabella: what the important stabilizer looks like in the Central European population. Biomed Res Int 2015, 1–8. 10.1155/2015/343728 PMC4564579 26413516

[joa12994-bib-0033] Hedderwick M , Stringer MD , McRedmond L , et al. (2017) The oblique popliteal ligament: an anatomic and MRI investigation. Surg Radiol Anat 39, 1017–1027. 28324130 10.1007/s00276-017-1838-7

[joa12994-bib-0034] Heideman GM , Baynes KE , Mautz AP , et al. (2011) Fabella fracture with CT imaging: a case report. Emerg Radiol 18, 357–361. 21305331 10.1007/s10140-011-0941-z

[joa12994-bib-0035] Hessen I (1946) Fabella: Sesamum genu superius laterale. Acta Radiol 27, 177–196. 21027362 10.3109/00016924609133785

[joa12994-bib-0036] Hou W (2016) Should we remove the fabella in total knee arthroplasy of osteoarthritis In: 5th International Conference of Orthopedic Surgeons and Rheumatology. pp. 74.

[joa12994-bib-0037] Houghton‐Allen BW (2001) In the case of the fabella a comparison view of the other knee is unlikely to be helpful. Australas Radiol 45, 318–319. 11531756 10.1046/j.1440-1673.2001.00928.x

[joa12994-bib-0038] Hukuda S , Mochizuki T , Ogata M , et al. (1983) The pattern of spinal and extraspinal hyperostosis in patients with ossification of the posterior longitudinal ligament and the ligamentum flavum causing myelopathy. Skeletal Radiol 10, 79–85. 6612370 10.1007/BF00360789

[joa12994-bib-0039] Ikeuchi H , Nagatsuka Y (1970) Fracture of the fabella, Seikeigeka. Orthop Surg 21, 559–563. 5464816

[joa12994-bib-0040] Itoman M , Yamamoto M , Okuda T , et al. (1976) Peroneal nerve palsy caused by compression of the fabella a case report. Orthop Traumatol 25, 322–325.

[joa12994-bib-0041] Jin ZW , Shibata S , Abe H , et al. (2017) A new insight into the fabella at knee: the foetal development and evolution. Folia Morphol 76, 87–93. 10.5603/FM.a2016.0048 27665955

[joa12994-bib-0042] Johnson JF , Brogdon BG (1982) Dorsal effect of the patella: incidence and distribution. Am J Roentgenol 139, 339–340. 6979889 10.2214/ajr.139.2.339

[joa12994-bib-0043] Jung U‐H , Chun C‐W , Park C‐S , et al. (2007) Arthroscopic treatment of fabella impingement syndrome after total knee arthroplasty ‐ a case report. J Korean Orthop Assoc 42, 832.

[joa12994-bib-0044] Kaneko K (1966) Einige Betrachtungen über die Fabella des Menschen. Acta Anat Nippon 42, 85–88.

[joa12994-bib-0045] Kato Y , Oshida M , Ryu K , et al. (2012) The Incidence and Structure of the Fabella in Japanese Population. Anatomical Study, Radiographic Study, and Clinical Cases. Long Beach, CA p. 1797.

[joa12994-bib-0046] Kawashima T , Takeishi H , Yoshitomi S , et al. (2007) Anatomical study of the fabella, fabellar complex and its clinical implications. Surg Radiol Anat 29, 611–616. 17882346 10.1007/s00276-007-0259-4

[joa12994-bib-0047] Kim T , Chung H , Lee H , et al. (2018) A case report and literature review on fabella syndrome after high tibial osteotomy. Medicine 97, e9585. 29369174 10.1097/MD.0000000000009585 PMC5794358

[joa12994-bib-0048] Kitahara M (1935) Röntgenuntersuchungen der Fabella bei Formosa‐Wilden. J Med Assoc Formosa 34, 533–543.

[joa12994-bib-0049] Kojima R (1958) Uber die Fabella bei den Japanern, insbesondere Dieselbe im hoheren Alter. Yokohama Med Bull 9, 339–347. 13604338

[joa12994-bib-0050] Kubota Y , Toyoda Y , Kubota H , et al. (1986) Common peroneal nerve palsy associated with the fabella syndrome. Anesthesiology 65, 552–553. 3777491 10.1097/00000542-198611000-00024

[joa12994-bib-0051] Kurtoğlu Z , Elvan Ö , Aktekin M , et al. (2015) A descriptive and morphometric study of the fabellofibular, arcuate popliteal and popliteofibular ligaments. Anatomy 9, 51–59.

[joa12994-bib-0052] Kuur E (1986) Painful fabella: a case report with review of the literature. Acta Orthop Scand 57, 453–454. 3811895 10.3109/17453678609014771

[joa12994-bib-0053] Kwee TC , Heggelman B , Gaasbeek R , et al. (2016) Fabella fractures after total knee arthroplasty with correction of valgus malalignment. Case Rep Orthop 20, 1–5. 10.1155/2016/4749871 PMC4908254 27340579

[joa12994-bib-0054] Laird L (1991) Fabellar joint causing pain after total knee replacement. J Bone Joint Surg. British volume. 73, 1007–1008. 10.1302/0301-620X.73B6.1955425 1955425

[joa12994-bib-0055] Larson JE , Becker DA (1993) Fabellar impingement in total knee arthroplasty. A case report. J Arthroplasty 8, 95–97. 8436997 10.1016/s0883-5403(06)80114-2

[joa12994-bib-0056] Lencina O (2007) Estudio anatómico y radiológico del sesamoideo del gemelo externo de la rodilla. Rev Asoc Argent Ortop Traumatol Año 72, 248–255.

[joa12994-bib-0057] Levowitz BS , Kletschka HD (1955) Fracture of the fabella: report of a case. J Bone Joint Surg 37, 879–7. 13242621

[joa12994-bib-0058] Loth E (1931) Anthropologie des Parties Molles: (Muscles, Intestins, Vaisseaux, Nerfs Périphériques). Warsaw: Mianowski Foundation Print Shop.

[joa12994-bib-0059] Lungmuss F (1954) Die Fabella, ihr Vorkommen und ihre Differentialdiagnose. Zentralbl Chir 15, 618–624. 13188261

[joa12994-bib-0060] Mangieri JV (1973) Peroneal‐nerve injury from an enlarged fabella: a case report. J Bone Joint Surg 55, 395–397. 4696171

[joa12994-bib-0061] Marks PH , Cameron M , Regan W (1998) Fracture of the fabella: a case of posterolateral knee pain. Orthopedics 21, 713–714. 9642711 10.3928/0147-7447-19980601-15

[joa12994-bib-0062] McElreath R (2016) rethinking: Statistical Rethinking book package.

[joa12994-bib-0063] Miaśkiewicz C , Partyka B (1984) Fabella in men of three human races. Folia Morphol 43, 369–374. 6336076

[joa12994-bib-0064] Minowa T , Murakami G , Kura H , et al. (2004) Does the fabella contribute to the reinforcement of the posterolateral corner of the knee by inducing the development of associated ligaments? J Orthop Sci 9, 59–65. 14767706 10.1007/s00776-003-0739-2

[joa12994-bib-0065] Minowa T , Murakami G , Suzuki D , et al. (2005) Topographical histology of the posterolateral corner of the knee, with special reference to laminar configurations around the popliteus tendon: a study of elderly Japanese and late‐stage fetuses. J Orthop Sci 10, 48–55. 15666123 10.1007/s00776-004-0848-6

[joa12994-bib-0066] Mohite S , Mohite H , More R , et al. (2016) Morphological study of the plantaris muscle. Int J Health Sci Res 6, 125.

[joa12994-bib-0067] Munshi M , Pretterklieber ML , Kwak S , et al. (2003) MR imaging, MR arthrography, and specimen correlation of the posterolateral corner of the knee: an anatomic study. Am J Roentgenol 180, 1095–1101. 12646462 10.2214/ajr.180.4.1801095

[joa12994-bib-0068] Nishimura H , Shimizu S (1963) Studies on various ageing phenomena in Japanese twins and siblings. Acta Genet Med Gemellol 12, 22–49. 13938780 10.1017/s1120962300016589

[joa12994-bib-0069] Okano E , Yoshioka T , Yanai T , et al. (2016) Fabella syndrome as an uncommon cause of posterolateral knee pain after total knee arthroplasty: a case report and review of the literature. Case Rep Orthop 2016, 1–5. 10.1155/2016/4328462 PMC4932162 27418991

[joa12994-bib-0070] Oransky M , Canero G , Maiotti M (1989) Embryonic development of the posterolateral structures of the knee. Anat Rec 225, 347–354. 2589648 10.1002/ar.1092250411

[joa12994-bib-0071] Ortega M , Olave E (2018) Presencia, localizacion y biometria de la fabela en individuos Chilenos: estudio radiologico. Int J Morphol 36, 358–362.

[joa12994-bib-0072] Orzincolo C , Scutellari PN , Aiello N , et al. (1987) Knee in diffuse idiopathic skeletal hyperostosis. Radiol Med, 74, 270–274.3671796

[joa12994-bib-0073] Osti M , Tschann P , Künzel KH , et al. (2013) Posterolateral corner of the knee: microsurgical analysis of anatomy and morphometry. Orthopedics 36, e1114–e1120. 24025000 10.3928/01477447-20130821-11

[joa12994-bib-0074] Pancoast H (1909) Radiographic statistics of the sesamoid in the tendon of the gastrocnemius. Univ Penn Bull 22, 213.

[joa12994-bib-0075] Parsons FG , Keith A (1897) Seventh Report of the Committee of Collective Investigation of the Anatomical Society of Great Britain and Ireland, 1896‐97. J Anat Physiol 32, 164–186. PMC1327966 17232286

[joa12994-bib-0076] Patel A , Singh R , Johnson B , et al. (2013) Compression neuropathy of the common peroneal nerve by the fabella. BMJ Case Rep 2013, bcr2013202154. 10.1136/bcr-2013-202154 PMC3847518 24293541

[joa12994-bib-0077] Pearson K , Davin AG (1921) On the sesamoids of the knee‐joint: part II. Evolution of the sesamoids. Biometrika 13, 350.

[joa12994-bib-0078] Phukubye P , Oyedele O (2011) The incidence and structure of the fabella in a South African cadaver sample. Clin Anat 24, 84–90. 20830786 10.1002/ca.21049

[joa12994-bib-0079] Piyawinijwong S , Sirisathira N , Sricharoenvej S (2012). The fabella, fabellofibular and short lateral ligaments: an anatomical study in Thais cadavers.

[joa12994-bib-0080] Pritchett JW (1984) The incidence of fabellae in osteoarthrosis of the knee. J Bone Joint Surg 66, 1379–1380. 6501334

[joa12994-bib-0081] R Core Team (2018) R: A Language and Environment for Statistical Computing. Vienna: R Foundation for Statistical Computing.

[joa12994-bib-0082] R Team (2015). R Studio: Integrated Development for R.

[joa12994-bib-0083] Raheem O , Philpott J , Ryan W , et al. (2007) Anatomical variations in the anatomy of the posterolateral corner of the knee. Knee Surg Sports Traumatol Arthrosc 15, 895–900. 17641923 10.1007/s00167-007-0301-4

[joa12994-bib-0084] Rankin I , Rehman H , Ashcroft GP (2018) Fabella syndrome following de‐rotation surgery to correct a femoral malunion. Open Orthop J 12, 346–352. 30197717 10.2174/1874325001812010346 PMC6118036

[joa12994-bib-0085] Robertson A , Jones SC , Paes R , et al. (2004) The Fabella: a forgotten source of knee pain? Knee 11, 243–245. 15194103 10.1016/S0968-0160(03)00103-0

[joa12994-bib-0086] Sagel J (1932) Fracture of sesamoid bones: a report of two cases. Am J Surg 18, 507–509.

[joa12994-bib-0087] Sarin VK , Carter DR (2000) Mechanobiology and joint conformity regulate endochondral ossification of sesamoids. J Orthop Res 18, 706–712. 11117290 10.1002/jor.1100180505

[joa12994-bib-0088] Sarin VK , Erickson GM , Giori NJ , et al. (1999) Coincident development of sesamoid bones and clues to their evolution. Anat Rec 257, 174–180. 10597342 10.1002/(SICI)1097-0185(19991015)257:5<174::AID-AR6>3.0.CO;2-O

[joa12994-bib-0089] Schönbauer HR (1956) Arthrose der Fabella. Fortschritte auf dem Gebiet der Röntgenstrahlen und der bildgebenden Verfahren. 85, 358–359. 13375756

[joa12994-bib-0090] Segal A , Miller TT , Krauss ES (2004) Fabellar snapping as a cause of knee pain after total knee replacement: assessment using dynamic sonography. Am J Roentgenol 183, 352–354. 15269024 10.2214/ajr.183.2.1830352

[joa12994-bib-0091] Sekiya JK , Jacobson JA , Wojtys EM (2002) Sonographic imaging of the posterolateral structures of the knee: findings in human cadavers. Arthroscopy 18, 872–881. 12368785 10.1053/jars.2002.32845

[joa12994-bib-0092] Seol P‐H , Ha KW , Kim YH , et al. (2016) Effect of radial extracorporeal shock wave therapy in patients with fabella syndrome. Ann Rehabil Med 40, 1124–1128. 28119844 10.5535/arm.2016.40.6.1124 PMC5256318

[joa12994-bib-0093] Silva JG , Chagas CAA , Torres DFM , et al. (2010) Morphological analyisis of the fabella in Brazilians. Int J Morphol 28, 105–110.

[joa12994-bib-0094] Stroup DF , Berlin JA , Morton SC , et al. (2000) Meta‐analysis of observational studies in epidemiology: a proposal for reporting. Meta‐analysis Of Observational Studies in Epidemiology (MOOSE) group. JAMA 283, 2008. 10789670 10.1001/jama.283.15.2008

[joa12994-bib-0095] Sudasna S , Harnsiriwattanagit K. (1990) The ligamentous structures of the posterolateral aspect of the knee. Bull Hosp Jt Dis Orthop Inst 50, 35–40. 2163702

[joa12994-bib-0096] Sutro CJ , Pomeranz MM , Simon SM (1935) Fabella (sesamoid in the lateral head of the gastrocnemius). Arch Surg 30, 777–783.

[joa12994-bib-0097] Tabira Y , Saga T , Takahashi N , et al. (2012) Influence of a fabella in the gastrocnemius muscle on the common fibular nerve in Japanese subjects. Clin Anat 26, 893–902. 22933414 10.1002/ca.22153

[joa12994-bib-0098] Takebe K , Hirohata K (1981) Peroneal nerve palsy due to fabella. Arch Orthop Trauma Surg 99, 91–95. 7316708 10.1007/BF00389743

[joa12994-bib-0099] Tang JY , Mulcahy H , Chew F (2010) High‐energy fracture of the fabella. Radiol Case Rep 5, 454–456. 27307881 10.2484/rcr.v514.454 PMC4901016

[joa12994-bib-0100] Tatagari V , Brehman E , Adams C (2018) Evaluation of the Gross Anatomical Incidence of Fabellae in a North American Cadaveric Population. Research Day.

[joa12994-bib-0101] Terry GC , LaPrade RF. (1996) The posterolateral aspect of the knee. Am J Sports Med 24, 732–739. 8947393 10.1177/036354659602400606

[joa12994-bib-0102] Theodorou SJ , Theodorou DJ , Resnick D (2005) Painful stress fractures of the fabella in patients with total knee arthroplasty. Am J Roentgenol 185, 1141–1144. 16247123 10.2214/AJR.04.1230

[joa12994-bib-0103] Uchino K , Nakamura H , Matuda G , et al. (1992) A case report of fabella syndrome assciated with rheumatoid arthritis. Orthop Traumatol 40, 1223–1224.

[joa12994-bib-0104] Upasna A , Nar A , Kumar A , et al. (2016) Morphological analysis of proximal gastrocnemius muscle – a study in thirty adult human cadavers. Int J Anat Radiol Surg 5, 41–43.

[joa12994-bib-0105] Urata Y , Okamoto S , Nakamura E , et al. (2015) A case of fabello‐femoral osteoarthritis (fabella OA) with locking‐like symptoms. Orthop Traumatol 64, 644–647.

[joa12994-bib-0106] Usmani S , Marafi F , Ahmed N , et al. (2017) 8F‐NaF PET‐CT in symptomatic fabella syndrome. Clin Nucl Med 42, e199. 28098667 10.1097/RLU.0000000000001547

[joa12994-bib-0107] Wang JW (1995) Fabellar impingement after total knee replacement – a case report. Changgeng yi xue za zhi 18, 185–189. 7641114

[joa12994-bib-0108] Weiner DS , Macnab I (1982) The ‘fabella syndrome’: an update. J Pediatr Orthop 2, 405–408. 6815224

[joa12994-bib-0109] Weiner D , Macnab I , Turner M (1977) The fabella syndrome. Clin Orthop Relat Res 126, 213–215. 598120

[joa12994-bib-0110] Wolf B , Bryk D (1959) Fabella arthritic changes. J Mt Sinai Med 26, 307–323. 13655053

[joa12994-bib-0111] Woo CC (1988) Fracture of the fabella. J Manipulative Physiol Ther 11, 422–425. 3235930

[joa12994-bib-0112] Yammine K (2014) The prevalence of the sesamoid bones of the hand: a systematic review and meta‐analysis. Clin Anat 27, 1291–1303. 24615762 10.1002/ca.22378

[joa12994-bib-0113] Yammine K (2015) The sesamoids of the feet in humans: a systematic review and meta‐analysis. Anat Sci Int 90, 144–160. 24801385 10.1007/s12565-014-0239-9

[joa12994-bib-0114] Yano K (1928) Das Os sesamoideum muscli gastrocnemii lateralis bei den Japanern. Folia Anat Japon 6, 241–246.

[joa12994-bib-0115] Yu JS , Salonen DC , Hodler J , et al. (1996) Posterolateral aspect of the knee: improved MR imaging with a coronal oblique technique. Radiology 198, 199–204. 8539378 10.1148/radiology.198.1.8539378

[joa12994-bib-0116] Zalasiewicz J , Waters CN , Ivar do Sul JA , et al. (2016) The geological cycle of plastics and their use as a stratigraphic indicator of the Anthropocene. Anthropocene 13, 4–17.

[joa12994-bib-0117] Zeng S‐X , Dong X‐L , Dang R‐S , et al. (2012) Anatomic study of fabella and its surrounding structures in a Chinese population. Surg Radiol Anat 34, 65–71. 21626275 10.1007/s00276-011-0828-4

[joa12994-bib-0118] Zenteno C , Morales C , De la Torre G (2010) Fabella syndrome in a high performance runner. Case presentation and literature review. Acta Ortopéd Mex 24, 264–266. 21305764

[joa12994-bib-0119] Zhou F , Zhang F , Deng G , et al. (2017) Fabella fracture with radiological imaging: a case report. Trauma Case Rep 12, 19–23. 29644278 10.1016/j.tcr.2017.10.010 PMC5887092

[joa12994-bib-0120] Zipple JT , Hammer RL , Loubert PV (2003) Treatment of fabella syndrome with manual therapy: a case report. J Orthop Sports Phys Ther 33, 33–39. 12570284 10.2519/jospt.2003.33.1.33

